# Urine-derived bladder cancer organoids (urinoids) as a tool for cancer longitudinal response monitoring and therapy adaptation

**DOI:** 10.1038/s41416-023-02494-6

**Published:** 2023-12-15

**Authors:** Bastiaan J. Viergever, Daniëlle A. E. Raats, Veerle Geurts, Jasper Mullenders, Trudy N. Jonges, Michiel S. van der Heijden, Johan H. van Es, Onno Kranenburg, Richard P. Meijer

**Affiliations:** 1 Laboratory Translational Oncology, Division of Imaging and Oncology, University Medical Center Utrecht, 3584CX Utrecht, The Netherlands; 2 Department of Oncological Urology, Division of Imaging and Oncology, University Medical Center Utrecht, 3584CX Utrecht, The Netherlands; 3 Utrecht Platform for Organoid Technology, Utrecht University, 3584 CX Utrecht, The Netherlands; 4 Oncode Institute, Hubrecht Institute, Royal Netherlands Academy of Arts and Sciences (KNAW) and University Medical Center Utrecht, 3584 CT Utrecht, The Netherlands; 5 Department of Pathology, University Medical Center Utrecht, 3584 CX Utrecht, The Netherlands; 6 Department of Medical Oncology, Antoni van Leeuwenhoek Hospital, 1066CX Amsterdam, The Netherlands

**Keywords:** Bladder cancer, Bladder cancer

## Abstract

**Background:**

Bladder cancer is one of the most common cancer types worldwide. Generally, research relies on invasive sampling strategies.

**Methods:**

Here, we generate bladder cancer organoids directly from urine (urinoids). In this project, we establish 12 urinoid lines from 22 patients with non-muscle and muscle-invasive bladder tumours, with an efficiency of 55%.

**Results:**

The histopathological features of the urinoids accurately resemble those of the original bladder tumours. Genetically, there is a high concordance of single nucleotide polymorphisms (92.56%) and insertions & deletions (91.54%) between urinoids and original tumours from patient 4. Furthermore, these urinoids show sensitivity to bladder cancer drugs, similar to their tissue-derived organoid counterparts. Genetic analysis of longitudinally generated tumoroids and urinoids from one patient receiving systemic immunotherapy, identify alterations that may guide the choice for second-line therapy. Successful treatment adaptation was subsequently demonstrated in the urinoid setting.

**Conclusion:**

Therefore, urinoids can advance precision medicine in bladder cancer as a non-invasive platform for tumour pathogenesis, longitudinal drug-response monitoring, and therapy adaptation.

## Background

Bladder cancer ranks among the top five and ten most common cancers worldwide in men and women, respectively [1]. Over 573.000 patients were diagnosed with bladder cancer worldwide in 2022 (IARC). Urothelial carcinoma (UCC) is the predominant histopathological subtype of bladder cancer (BC) and on initial staging presents as non-muscle-invasive bladder cancer (NMIBC; 73% of total) or muscle-invasive bladder cancer (MIBC) [2–4]. In general, NMIBC has a high recurrence rate (up to 84%), but rarely metastasizes, whereas MIBC is an aggressive disease with a high risk for relapse and metastases (up to 50%) [3, 4]. Current neoadjuvant cisplatin-based combination chemotherapy regimens result in a complete pathological response in around 25% of the cases [5]. Thus, evaluation of tumour biology and assessment of chemosensitivity of the individual bladder tumour is needed to guide personalised bladder cancer treatment [6–11]. Large-scale genetic analyses of bladder cancer have identified drivers such as *TP53, ARID1A, PIK3CA, FGFR3, STAG2* and *ERBB2* and have yielded several molecular classification methods [12], with consensus markers such as TP63 (Transitional/Intermediate cells), KRT5 (Basal class), KRT20 (Luminal class) and UP3A (Urothelial differentiation) [11, 13–16]. However, it remains challenging to apply such information in clinical practice to guide treatment decision-making [17]. In addition, drug treatment itself may induce changes in tumour characteristics, such as genetic instability, and cause enrichment of specific molecular subclones, which may result in altered drug sensitivity and acquired drug resistance [17–20]. Platforms that allow drug-response monitoring longitudinally may therefore have great value in developing personalised and adaptive treatment strategies.

Recent developments in new technologies allow to grow living three-dimensional (3D) tumour structures on a patient-specific base (organoids). These in vitro multicellular 3D structures resemble key features of their original tumour tissue, are self-organising and self-renewing. Thereby, organoids allow in vitro patient-specific tumour analysis and screening [21–24]. Organoids derived from bladder tumour tissue (surgical biopsy) reflect the heterogeneity and molecular subclassification of the corresponding original tumour samples and can be used as a platform for testing drug sensitivity [16]. Urine offers a potential alternative source for generating organoids from the urinary tract. Previously, benign kidney- and urothelium-derived organoids have been established from urine of healthy individuals [16]. Importantly, urine from bladder cancer patients generally contains exfoliated viable tumour cells, providing a potential source for non-invasive tumour sampling [4].

In this proof-of-concept study, we investigate whether urine-derived tumour cells can be used for the generation of organoids (‘urinoids’) from bladder cancer patients. In addition, we test the hypothesis that urinoid generation provides an effective non-invasive strategy for tumour subtyping and longitudinal drug-response monitoring. We show that a single urine sample from a bladder cancer patient can be used as non-invasive sampling method for the generation of bladder cancer organoids with 55% efficiency. We show that these urinoids recapitulate histopathological features and genetic mutational status of the original tumour tissue. We show that urinoid clonality is similar to matched tumour tissue-based organoids (‘tumoroids’). Furthermore, we use both tumoroids and urinoids to identify the increase of a patient-specific structural variations after immunotherapy. We find a significant increase of mutational burden in among others microtubule-based processes and de novo chromoplexy events in both the urinoids and tumoroids after immunotherapy. Finally, we find that both urinoid and tumoroid lines after immunotherapy show a significant increase of sensitivity to the microtubule targeting agents vinblastine and vincristine, which is not seen in the respective pre-immunotherapy tumoroids. Overall, we conclude that urinoids are a non-invasive tool for following tumour pathogenesis, longitudinal drug-response monitoring, and therapy adaptation in bladder cancer.

## Methods

### Approval of studies involving human tissue and patient-inform consent

Bladder cancer patients from the University Medical Center Utrecht were invited to participate in this prospective proof-of-concept project by means of informed consent by the treating urologist or the nurse practitioner. Human bladder tissue was obtained from the University Medical Center Utrecht (UMCU). Ethical approval was granted by the Biobank Research Ethics Committee (TCBio) of the UMCU. Written informed consent was obtained from all patients involved in this project. Bladder tissue was obtained through transurethral bladder tumour resections (TUR-BT) or cystectomy procedures. Urine samples were obtained through transurethral catheterisation at the time of surgery. All samples were examined by a dedicated uropathologist.

### Human bladder organoids establishment and culture

Human bladder tissue was examined and selected for malignancy by dedicated uropathologists. In both the TUR-BT and the cystectomy cases, we obtained a sample of tumour tissue from the patient. The tissue was cut into smaller pieces (1 mm to 2 mm) with a surgical blade, of which half was frozen as the original tumour sample. The remaining half was digested with Liberase (Sigma-Aldrich, 5401135001) in Advanced DMEM/F-12 (ThermoFisher, 12634028) with ROCK inhibitor (Y-27632, 10 μM) for 60 min at 37 °C. This resulted in the generation of tissue-based organoids called *tumoroids*. Urine samples were collected at the start of the TUR-BT or cystectomy operation via catheterisation into 25 mL DPBS (Corning Life Sciences, 21-031-CVR), with urine volume ranging from 5 to 75 mL samples. Urine tumour cells were collected by centrifugation and washed with DPBS for a minimum of five times. After centrifugation, the cell pellet was resuspended in ∼200 μL of RGF BME (R&D Systems Europe, 3533-010-02) and plated into one to two individual wells of a prewarmed six-well plate. This resulted in the generation of urine-based organoids called urinoids. In both tissue- and urine-based establishments, when the BME was solidified, human bladder organoid media was added. Human bladder organoid media consisted of Advanced DMEM/F-12, FGF10 (100 ng/mL of Peprotech 100-26), FGF7 (25 ng/mL of Peprotech 100-19), FGF2 (12.5 ng/mL of Peprotech 100-18B), B27 supplement (Fisher Scientific, 11530536), A83-01 (5 μM), N-acetylcysteine (1.25 mM), and nicotinamide (10 mM). Human bladder organoids were passaged weekly and either sheared through a pipette or by dissociation using TrypLE (Fisher Scientific, 12604021). ROCK inhibitor (Y-27632, 10 μM) was added to the media after passaging, to prevent passaging-induced cell death. Organoids were frozen in Recovery^TM^ Cell culture Freezing Medium (Fisher Scientific, 12648010) and stored in the UMCU Central Biobank (CBB).

### Immunohistochemistry

Organoids and tissue were fixed in 4% paraformaldehyde for 1–6 h, dehydrated, and paraffin-embedded according to standard histology procedures. Sections were stained with Hematoxylin & Eosin (HE) or the following antibodies: Keratin 5 (Abcam, ab52635), Ki67 (Dako, M7240), TP53 (Santa Cruz, sc-126), TP63 (Abcam, ab735) and Uroplakin III (SFI-1, Abcam, ab78196) according to the manufacturer’s protocols. For Keratin 20 (DakoCytomation, M7019), EDTA antigen retrieval was used in combination with a Ultravision Protein Block (Fisher Scientific, TA-125-PBQ) for 30 min at room temperature. The following secondary antibodies conjugated with HRP were used: bright vision poly-hrp-anti-rabbit igg (VWR international, VWRKDPVR110HRP) and bright vision poly-hrp-anti-mouse igg (VWR international, VWRKDPVM110HRP) for 1 h. at room temperature, and Goat IgG HRP-conjugated Antibody (R&D Systems Europe, HAF017) for 1 h. at 37 °C. Staining was performed using 3,3’-diaminobenzidine (DAB) for 10 min exactly at room temperature. Images were acquired by high-resolution scanning of the slides and analysed using the NDP.view (v2.7.39) software (Hamamatsu Photonics K.K.). These sections were evaluated in a blinded test by dedicated uropathologists. Regrettably, due to a clerical error, the original tissue of UBTOR5 was not available for immunohistochemical analysis and thus excluded for the comparison.

### DNA isolation and library preparation

DNA was isolated using the QIAamp® DNA mini kit according to the manufacturer’s instructions. Per sample, 500–1000 ng of DNA was used for DNA library preparation. Library preparation was performed following the Truseq DNA nano protocol.

### Whole-genome sequencing

Whole-genome sequencing was performed by the Utrecht Sequencing Facility using the Illumina NovaSeq 6000 set-up and analysed using the nf-core/sarek pipeline (v2.7.1) with reference genome assembly GRCh38 and with the GRIDSS-PURPLE-LINX pipeline (v1.3.2). Paired-end whole-genome sequencing was performed with an average coverage of 30x. For both pipelines, normal-tumour mode could not be used, but all modules were run with tumour-only, single-sample and/or multiple-sample variant calling workflows when possible.

### Single-cell karyotype sequencing

Single-cell karyotype sequencing was provided by Single-Cell Core of the Oncode Institute, Utrecht, the Netherlands [25]. Nuclei in 384-well plates are digested with NlaIII, after which the genomic fragments (following end processing) are ligated to barcoded-adaptors containing a unique molecular identifier (UMI), cell-specific barcode, and T7 promoter allowing linear amplification by in vitro transcription (IVT). Libraries were sequenced on an Illumina Nextseq500 with 2×75-bp paired-end sequencing. The data preprocessing has been performed using the SingleCellMultiOmics package developed at the Van Oudenaarden lab (https://github.com/BuysDB/SingleCellMultiOmics) and the sequences were mapped to the homo sapiens genome (Ensembl, version 97) using Burrows-Wheeler Aligner (BWA). The heatmap and consensus plots were produced using the Aneufinder library (https://bioconductor.org/packages/release/bioc/html/AneuFinder.html) using a step size of 5e + 5 with the edivisive algorithm.

### Drug response

Organoids were split using TrypLE, 3–4 days before experiments, organoids were made single cells. On the day of the experiment, organoids were harvested, strained through a 70-μm filter and counted, after which 2000 organoids per well were plated in a 96-well plate in culture media containing 5% BME. Medium composition is altered due to screening of platinum-based drugs [26], using Dulbecco’s Modified Eagle’s Medium/Nutrient Mixture F-12 Ham with the respective additives as described in the culture section of this materials and methods with the adjustment of omitting N-acetylcysteine. One day later, drugs were added at the indicated concentration and cells were incubated for 96 hours. Cell viability was measured using CellTiter-Glo 3D (Promega, G9683) according to the manufacturer’s instructions using a Spectramax.

### Data analysis and statistics

Data processing, analysis and visualisation were performed using R studio (Build 554; 2022.07.1). SNP enrichment analysis was performed using the gProfiler2 package (v0.2.1) using a false discovery rate (FDR) with a significance cut-off (*P* < 0.05). gProfiler2 results were visualised using the R package enrich-plot (v1.16.2) as part of the clusterProfiler tool package [27]. Drug screen results were quantified using GraphPad Prism (v9.4.0) consisting of three biological and two technical replicates analysed with a two-way ANOVA Tukey’s multiple comparisons significance test performed on the Area Under the Curve (AUC) data. AUC data was generated using the Area Under Curve function of GraphPad Prism on the Mean and Standard error of Mean (SEM) data from the baseline corrected medium only control ATP reads. ANOVA Tukey’s multiple comparisons significance test settings, values and results are summarised in Supplemental Table 4. Significance indicated correspond with ^ns^*P* value > 0.1234, *P < 0.033, **P < 0.0021, ***P < 0.0002 and ****P < 0.0001.

## Results

### Urine as a source for non-invasive generation of bladder cancer organoids

**Fig. 1:**
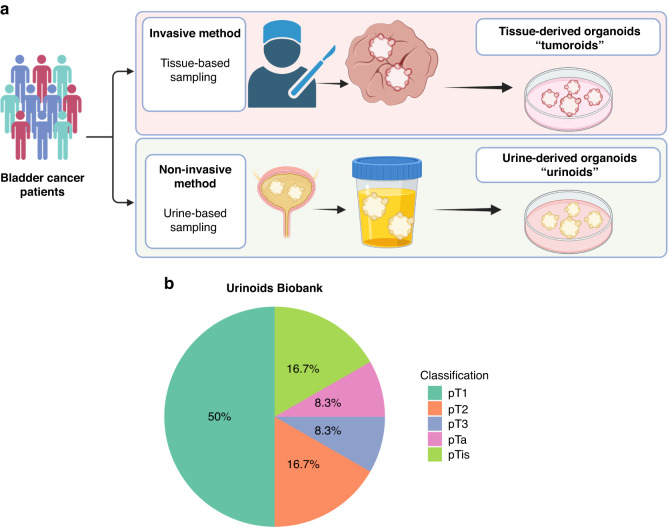
Bladder urinoid and tumoroid application and establishment. **a** Schematic representation of non-invasive organoid sampling for bladder cancer patient stratification. **b** TNM classification distribution pie-chart overview of all urinoids generated in this project, a summary of Table 1 and Supplemental Table 2.

Bladder cancer patients from the University Medical Center Utrecht were invited to participate in this prospective proof-of-concept project by means of informed consent by the treating urologist or the nurse practitioner. Tumour tissue biopsies and urine samples were obtained from consenting bladder cancer patients, who underwent transurethral bladder tumour resection (TUR-BT) or a radical cystectomy. The urine samples were collected using a transurethral catheter (Fig. 1). Urine-derived cells were subsequently cultured in a bladder cancer organoid medium to generate organoids (urinoids) [16]. In parallel, bladder cancer organoids were generated from resected tumour tissue (tumoroids), which were also fresh-frozen for histopathological and genetic analyses. In total, 22 bladder cancer patients were included in this study, resulting in a living biobank of 12 urinoid lines (establishment efficiency 55%; Fig. 1b, Table 1 and Supplemental Table 1). Urinoids were generated from patients with various disease stages including NMIBC (Ta (8.3%); CIS (16.7%); T1 (50%)) and MIBC (T2 (16.7%); T3 (8.3%)) (Fig. 1b, Table 1 and Supplemental Table 1).

**Table 1:** Overview of all urinoids generated in this project giving an overview of all patients.

Patient #	Line	Classification	Subclassification	Gender	Passage number
4	UBTOR4.2	MIBC	ypT1G3 Papillary UCC	M	P16
5	UBTOR5	MIBC	pTisN0MxR0	F	P13
6	UBTOR6.1	NMIBC	pT1G3 Papillary UCC	F	P6
7	UBTOR7.1	NMIBC	pT1G3 Papillary UCC	M	P10
8	UBTOR8	MIBC	pTisN0MxR0	M	P5
21	UBTOR21	MIBC	pT3N0MxR0 SCC + PI	M	P11
36	UBTOR36.2	NMIBC	pT1G2-3 Papillary UCC	M	P7
36	UBTOR36.3	NMIBC	pT1G2-3 Papillary UCC	M	P5
38	UBTOR38	NMIBC	pTaG1-2 Papillary UCC	M	P5
41	UBTOR41	MIBC	pT2bG3N0 UCC	M	P8
45	UBTOR45	MIBC	pT2G3 UCC	M	P7
57	UBTOR57	NMIBC	pT1G3 Papillary UCC	M	P5

*UCC* urothelial carcinoma, *SCC* squamous cell carcinoma, *PI* peritoneal invasion.

Table overview of all urinoids generated in this project giving an overview of all patients, urinoids generated, TNM classification, pathological stage of the tumour, gender and passage of the organoid line. All lines were stocked at lower passage numbers than the indicated passage number. Lines were taken out of long-term culture at indicated passage number.

### Urinoid cultures capture the histopathological features of the original tumour tissue

**Fig. 2:**
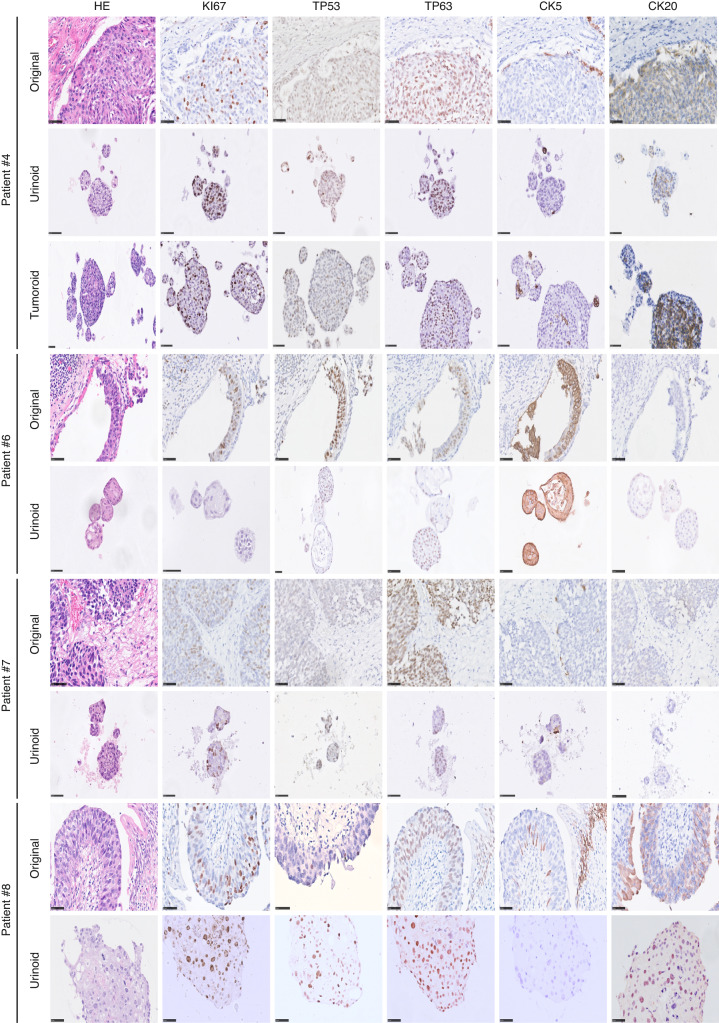

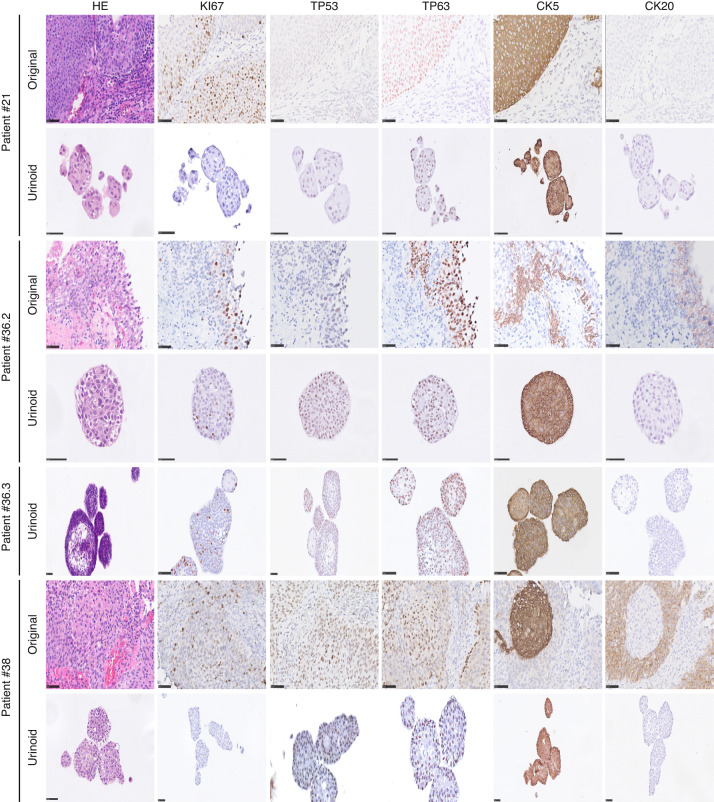
Immunohistochemical analysis of bladder urinoids. Immunohistochemical analysis of original tumour tissues, urinoids and tumoroids displayed with hematoxylin and eosin (HE) and their expression of Ki67, TP53, TP63, Keratin 5 (CK5) and Keratin 20 (CD20). Scale bars are 50 µm for tissues and organoids slides.

All urinoids, paired tumoroids and the original non-cultured tumour tissues were analysed by hematoxylin–eosin (HE) staining and by immunohistochemistry (IHC) to detect expression of bladder cancer subtype-specific and proliferation markers (Fig. 2 and Supplemental Fig. 1a–c). HE staining revealed that the histopathological morphology of the original tumour tissues was recapitulated in both the respective tumoroid and urinoid cultures (Fig. 2 and Supplemental Fig. 1c). IHC analysis of Ki67 by the pathologist determined that the percentage of positive (proliferative) tumour cells was highly similar among the original tumour tissues and paired tumoroids and urinoids (Fig. 2). Furthermore, expression of p53, p63, CK5 and CK20 was highly concordant among the original tumour tissues and paired tumoroids and urinoids (Fig. 2 and Supplemental Table 2). All original tissues, tumoroids and urinoids were negative for the nuclear kidney marker PAX8 (Supplemental Fig. 2). All tumour tissues and organoid lines were negative for the urothelial differentiation marker uroplakin 3a (UP3A)(Supplemental Fig. 2), which occurs in urothelial cancers [28, 29]. Urinoids were passaged for up to one year (~15 passages) and successfully biobanked via cryopreservation as a resource for future studies. Successful urinoid generation was not related to tumour stage (Supplemental Table 1). In several patients (*n* = 3), during surgery, malignancy was suspected, but upon further histopathological investigation revealed only benign tissue (pT0N0). Attempts for urinoid and tumoroid establishment from these patients all failed, highlighting the selectivity of the organoid growth medium for bladder cancer cells.

### Mutational status of the urinoids resemble original tumour tissue of patient 4

**Fig. 3:**
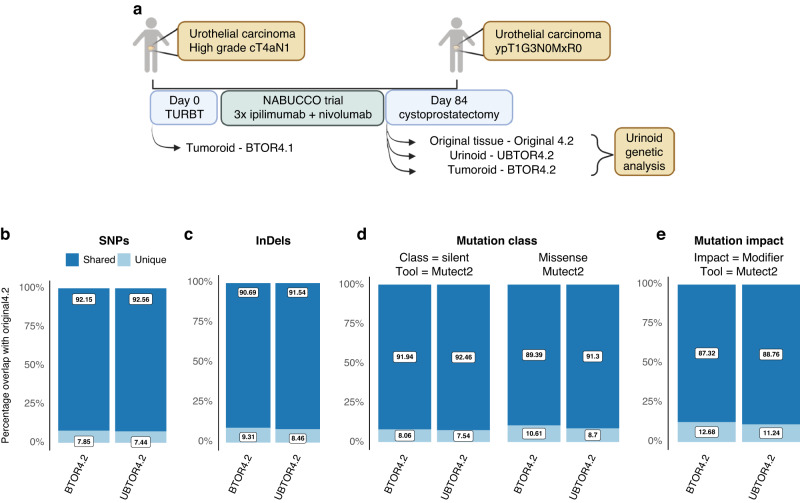
Urinoid UBTOR4.2 & tumoroid BTOR4.2 share high percentages of mutations with original tumour sample 4.2. **a** Patient 4 treatment schedule and sampling overview for whole-genome sequencing samples. Overlap of post-treatment (4.2) organoid lines shared with or unique mutations in respect to the original tumour sample 4.2 in **b** SNPs, **c** InDels, **d** modifier impact mutations, **e** overlap of silent and missense class mutations using Mutect2 tools from the Sarek pipeline.

Genetic resemblance of urinoids to the original tumour tissue was studied using bulk whole-genome sequencing (WGS). For this in-depth genetic analysis, samples from patient 4 were analysed. This patient was selected, as the establishment of both urinoids and tumoroids were successful and this patient underwent a novel immunotherapy treatment due to platinum-based therapy ineligibility, as part of the NABUCCO-trial, which consisted of three cycles of ipilimumab (1 mg/kg) and nivolumab (3 mg/kg) (Fig. 3a) [30]. At the start of this trial, patient 4 was diagnosed with a high-grade cT4aN1 invasive bladder cancer and suspected of invasion of the prostate. At the transurethral bladder tumour resection (TUR-BT), a tumour tissue sample was collected and a pre-immunotherapy tumoroid line (BTOR4.1) was generated. The patient had a radiological reduction in tumour size (53 to 21 mm diameter) in response to the pre-operative immunotherapy treatment, with no signs of invasion of the prostate. This resulted in a post-therapy TNM of ypT1G3. Subsequently, a radical cystoprostatectomy was performed. At the cystoprostatectomy, samples were harvested from the tumour for both the unaltered post-immunotherapy baseline tumour sample, the original tumour sample (Original 4.2), and for the establishment of both post-immunotherapy urinoid (UBTOR4.2) and tumoroid (BTOR4.2) lines.

All urinoid and tumoroid samples of patient 4 were analysed by WGS, to evaluate genetic resemblance to the original bladder tumour. The mutational status of bladder cancer driver genes in urinoid and tumoroids samples are found to be identical to those found in the original tumour 4.2 (Supplemental Table 5). Known bladder oncogenes of the original tumour were all also altered in UBTOR4.2. Interestingly, *GATA3* mutations were not detected in BTOR4.1 as well as a low impact alteration in3 *KRT5* for BTOR4.2 compared to both UBTOR4.2 and the original tumour 4.2. Furthermore, *KRT20* is mutated with a splice region variant and intron variant in BTOR4.2, which is not present in the original tumour 4.2 nor UBTOR4.2. Further similarity of all post-therapy samples was based on single nucleotide polymorphisms (SNPs). Highly similar counts of SNPs were observed from the urinoid UBTOR4.2 and tumoroid BTOR4.2 (Supplemental Fig. 3a) when compared to the original tumour sample 4.2. SNP similarity was further confirmed by correlating the individual SNPs to those in the original tumour tissue. SNPs were found to be either unique to the organoid lines or shared with the original tumour tissue (Fig. 3b). A high degree of shared SNPs were found for UBTOR4.2 (92.56%) and BTOR4.2 (92.15%), compared to the original tumour sample 4.2. Next, the absolute number (Supplemental Fig. 3b) and the degree of shared (Fig. 3c) insertions and deletions (InDels) were analysed. Similar absolute counts and a high degree of shared InDels were observed between UBTOR4.2 (91.54%), BTOR4.2 (90.69%) with original tumour sample 4.2. The predicted class and impact of mutations were analysed with the snpEff tool. All post-immunotherapy organoids were highly similar to the original tumour sample 4.2 in counts (Supplemental Fig. 3c) and relative degree of shared missense and silent mutations (Fig. 3d). Interestingly, higher percentile variance was found in the nonsense mutations (Supplemental Fig. 3e). These were disregarded as significant differences because of the low absolute counts of nonsense mutations. The predicted impact variance of all SNPs and InDels on affected genes was found mainly in modifying gene impact mutations (Supplemental Fig. 3d). The degree of shared modifier (Fig. 3e), low, moderate and high impact mutations (Supplemental Fig. 3f) was high for all post-therapy organoids compared to the original tumour sample 4.2. Interestingly, high similarity of SNPs, InDels, class and impact of mutations were found in the pre-immunotherapy tumoroid line BTOR4.1 versus the post-immunotherapy original tumour sample 4.2 (Supplemental Fig. 3g–j, respectively). This suggests that any difference between pre- and post therapy should be found within the smaller unique percentages or outside of the SNP and Indel mutations. Overall, urinoids from patient 4 were found to be highly similar on the genetic level compared to the original tumour tissue.

### Clonality in urinoid cultures similar to tissue-based tumoroids

To determine if urine-based organoid establishment selects for genetic clonal lines, single-cell karyotype sequencing [25] was performed for BTOR4.1, BTOR4.2 and UBTOR4.2 (Supplemental Fig. 4a–c, respectively). Though several single cells show copy number variations (CNVs), the consensus of the cells for each line is diploid (Supplemental Fig. 4a–c, bottom plots). Only larger CNVs were found in the Y-chromosome, which is often disregarded [25]. Diploid bladder tumours have previously been reported in several bladder cancer cell lines and patients [31, 32]. Clustering all cells using a principal component analysis (PCA), most cells cluster together for all organoid lines (Supplemental Fig. 4d, e). All organoid lines derived from patient 4 showed few CNVs and no genetic subclones within the organoid populations. Overall, no treatment induced large copy number variations were detected. Furthermore, no subclones were introduced by the urine-based organoid establishment.

### Increase of structural variations in patient 4 after immunotherapy

**Fig. 4:**
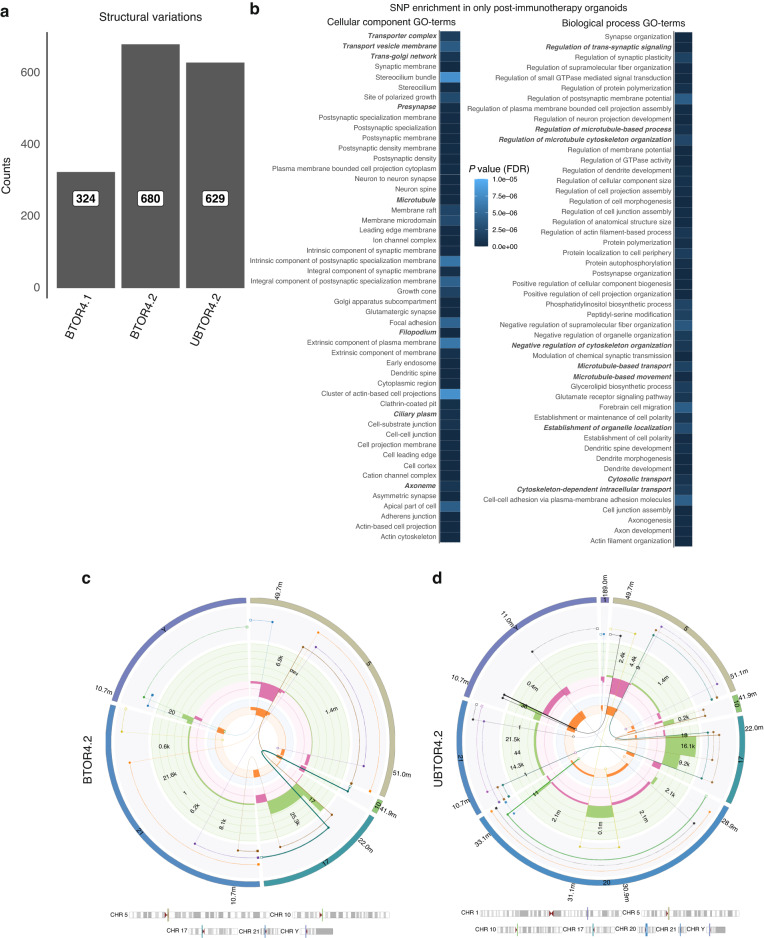
Immunotherapy induced structural variations, microtubule defects and chromoplexy in patient 4. **a** Patient 4 total structural variation counts of BTOR4.1: tissue-derived tumoroids patient 4 before immunotherapy; BTOR4.2 tissue-derived tumoroids patient 4 post immunotherapy and UBTOR4.2 urinoids post immunotherapy from the GRIDSS-PURPLE-LINX pipeline. **b** Highest quality scoring SNP enrichment terms (*n* = 50) of SNPs only found in post-treatment organoids affecting genes associated with GO terms of Cellular Components (CC, left) and Biological Process (BP, right), significance using false discovery rate (*P* < 0.05), bold italic highlighted terms are associated with microtubules. Complex multichromosomal linked structural variations as found by the LINX analysis of the GRIDSS-PURPLE-LINX pipeline for **c** tumoroid BTOR4.2 on chromosomes 5, 10, 17, 21 & Y and **d** urinoid UBTOR4.2 on chromosomes 1, 5, 10, 17, 20, 21 and Y.

Using the GRIDSS-PURPLE-LINX whole-genome analysis pipeline, larger mutations and overall genetic make-up were analysed in all organoid lines derived from patient 4. Based on the CNV and B-allele frequency (BAF) (Supplemental Fig. 5a–c), the majority of the genome was found to be diploid copy numbers with only several smaller CNVs. This confirms previous findings of single-cell karyotype sequencing. Interestingly, differences between pre- and post immunotherapy were found when studying the structural variations (SVs). The absolute number (Fig. 4a) of structural variations (SVs) in the organoid lines of patient 4 show an increase of SV counts in both the post-immunotherapy organoid lines UBTOR4.2 and BTOR4.2. This increase in SVs might be caused due to a recent catastrophic genetic rearrangement [33–35]. Studying the fusion genes found in BTOR4.1, BTOR4.2 and UBTOR4.2 (Supplemental Table 3), gene fusion *FGFR3-TACC3* was found in all lines. *FGFR3-TACC3* fusion has been previously linked to a poor prognosis and bladder cancer tumour progression [36]. Interestingly, a small decrease of fusion proteins was detected in the post-immunotherapy organoids BTOR4.2 (*n* = 8) and UBTOR4.2 (*n* = 8), compared with the pre-immunotherapy BTOR4.1 (*n* = 11) (Supplemental Table 3).

### Increase of SNP mutational burden in microtubule-based processes in patient 4 after immunotherapy

It was hypothesised whether further genetic differences between the pre- and post-immunotherapy organoid lines could be found. As SNP counts and overlap are similar, SNPs only found in both post-immunotherapy lines were analysed using an unbiased SNP pathway enrichment analysis (Fig. 4b and Supplemental Fig. 5d). Taking the top 50 quality SNP enrichments of all Gene Ontology (GO) segments, significant enrichment of SNPs (*P* < 0.05) was found in (among others) pathways relying on the microtubule network and other key mitotic processes. Previously, similar defects in microtubule-based and mitotic processes were shown to cause defects in structural stability and induce large chromosomal copy number variations [37, 38].

### Chromoplexy events in patient 4 after immunotherapy

As genetic differences between pre- and post immunotherapy were found in SVs, the unique SVs in both UBTOR4.2 and BTOR4.2 were studied further. Several complex link SV rearrangements were observed in both tumoroid BTOR4.2 and urinoid UBTOR4.2 (Fig. 4c, d and Supplemental Fig. 5e–g) [39]. This complex multichromosomal (> 2) linked SVs have previously been documented as variant of chromoanagenesis called ‘chromoplexy’ in oesophagus and prostate cancer [40, 41]. No multichromosomal complex-linked SVs were found in BTOR4.1. Overall, these results indicate that a chromoplexy event emerged during the ipilimumab and nivolumab immunotherapy of patient 4.

### Increased sensitivity to microtubule destabilising agents after immunotherapy and chromoplexy events

**Fig. 5:**
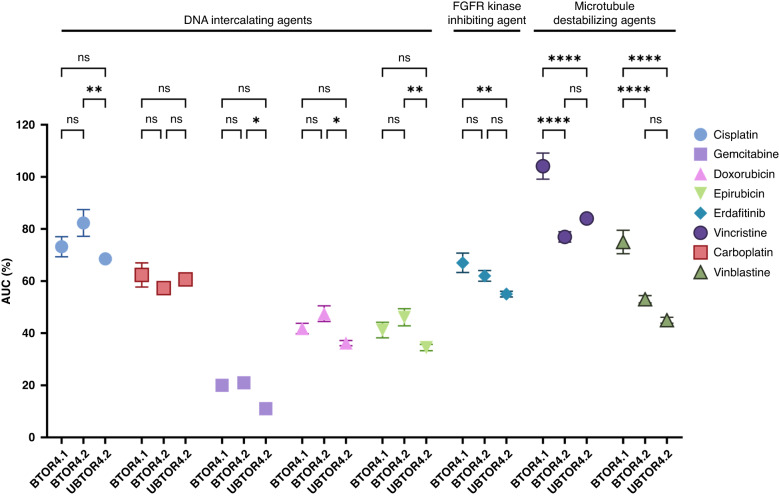
Application of urinoids in bladder cancer patients drug stratification. Area under the curve (AUC) values of the each drug depicted in Supplemental Fig. 7 with s.e.m. variation. Significance is calculated based on the AUC by two-way ANOVA with significance indicated with ^ns^*P* > 0.1234, *P < 0.0332, **P < 0.0021, ***P < 0.0002 and ****P < 0.0001 with settings and each seperate value depicted in Supplemental Table 4.

The drug responses of the longitudinally generated urinoids and tumoroids from patient 4 were evaluated, by exposure to a variety of commonly used DNA-intercalating drugs (cisplatin, gemcitabin, carboplatin, doxorubicin, epirubicin), microtubule destabilising agents (vinblastine, vincristine) and a novel fibroblast growth factor kinase inhibiting agent (erdafitinib) (Fig. 5 and Supplemental Fig. 6a–h). Erdafitinib was tested given the *FGFR3-TACC3* fusion and to evaluate urinoid application for novel drug efficacy evaluation. In particular, the microtubule destabilising agents were tested based on the discovered increased SNP load in microtubule-based processes in all post-immunotherapy organoids. The relative sensitivity per drug concentration was summarised in total areas under the curves (AUCs), measured using the relative ATP levels (Fig. 5). Overall limited responses were found for cisplatin (75%), carboplatin (60%) and erdafitinib (65%) in all organoids derived from patient 4, supporting the diagnosed platinum-based drug ineligibility. Moderate responses were found to doxorubicin (45%) and epirubicin (45%), and overall high sensitivity to gemcitabine (17.5%). Interestingly, a highly significant difference in sensitivity to microtubule destabilising agents vincristine (*P* < 0.0001) and vinblastine (*P* < 0.0001) was seen between pre-treatment BTOR4.1 and both post-treatment lines BTOR4.2 and UBTOR4.2. Overall, these results underline the potential of urinoids as non-invasive method to evaluate drug response in bladder cancer patients during treatment.

## Discussion

This proof-of-concept study demonstrates that non-invasive patient-derived urine-based bladder cancer organoids (urinoids) can be successfully generated from urine samples. These urinoids recapitulate the histopathological features of the original tumour tissue, such as degree of proliferation and molecular subtype differentiations, found in both patients with non-muscle-invasive and muscle-invasive bladder cancer. In-depth genetic analysis shows that mutational profiling of the urinoids closely resembles between the original tissue and the correlating tissue-based tumoroids (patient 4). Finally, the urinoids respond similarly to their respective tissue-based tumoroids in drug efficacy evaluation of standard and novel bladder cancer therapies.

Of special interest is the difference in the SNP load that was found post-compared to pre-immunotherapy. The post-immunotherapy urinoid and tumoroid lines contain a significantly enriched SNP load in the mitotic machinery and microtubule network, compared to pre-immunotherapy samples. This increased SNP load could have caused an increase in structural variations and a catastrophic chromoplexy event, which were detected in both of the post-immunotherapy urinoid and tumoroid lines [38]. Previously observed chromoplexy has not yet been directly linked to a specific moment or treatment in a singular patient [40–43]. As a significant increase of SNP load was found in microtubule-based processes, drug sensitivity to two microtubule destabilising agents was evaluated. Compared to the pre-immunotherapy tumoroid line, both post-immunotherapy chromoplexic urinoids and tumoroids showed a significant increase in sensitivity to microtubule targeting agents (i.e., vincristine and vinblastine). These drug-response results support the de novo increase of the SNP load in the microtubule-based processes for both post-immunotherapy urinoid and tumoroid lines of patient 4. We hypothesise that these mutations lead to both de novo increased SVs and targeted treatment sensitivity that occured in the short timeframe of patient 4’s 84 day treatment window. Whether these de novo increase of SNPs is immunotherapy induced or due to a treatment selection on already present mutations in a small clonal population could not be determined. Defective mitotic machinery is often the cause for chromosomal rearrangements with chromosomal instability [25, 44–47]. Further specialised studies are needed to understand the underlying mechanisms of these chromoplexy events in the context of this immunotherapy. If this event is found to be common, the combination of immunotherapy with microtubule destabilization drugs (e.g., vincristine and vinblastine) could potentially have a synergistic therapeutic effect. Furthermore, patient 4’s platinum-based therapy ineligibility was confirmed in all patient 4 tumoroid and urinoid lines, with relative AUCs of cisplatin (75%) and carboplatin (60%) treatment respectively. Overall, this proof-of-concept study shows that urinoids can be generated for in vitro monitoring and drug-response prediction in bladder cancer. Both urine-derived and tissue-derived post-immunotherapy organoids showed specific treatment-induced de novo drug sensitivity for microtubule destabilization drugs. This similarity in sensitivity highlights the feasibility and value of sequential (follow-up) monitoring of a patient’s specific bladder tumour characteristics using the non-invasive urinoid approach. The currently reported urinoid establishment success rate of 55% has already led to the following practical improvements: (I) collecting more than one single urine sample, (II) urine collection in culture medium instead of PBS and (III) the addition of antibiotics to collection volume.

### Clinical applications, challenges and limitations of urinoids

Due to the unique non-invasive sampling method, urinoids can be implemented earlier in patient treatment pathways and are less burdensome for patients compared to tissue-based tumoroid approaches. This provides the opportunity to evaluate the specific tumour genetic make-up, therapy sensitivity and therapy resistance sooner. With rapid-organoid-based explant techniques, such as MOS [48] or Explant [49], within weeks urinoids provide a non-invasive tool for patient-specific drug screening, and for the monitoring of all stages of urothelial cancer, tumour progression and recurrence.

Besides the benefits of the non-invasive sampling of urinoids, there are certain challenges and limitations: (1) the urine-based approach results in a higher chance of culture infection due to voluntary urine sampling in a non-sterile setting when compared to the sterile operation room setting for tissue samples. In ten out of twenty-two patients the urinoid cultures were infected with bacteria or yeast. This problem has subsequently been addressed with the addition of specific antibiotics and antimycotics to the culture medium and doubling the number of samples per patient. These changes will increase the culturing efficiency. (2) both urinoids and tumoroids cultures lack tumour microenvironment (TME) components. Several ways have been established that allow the representation of the tumour microenvironment components, such as coculture methods with stromal or immune components. This is especially relevant when rapid-organoid-based explant techniques are used (such as MOS [48] or Explant [49]), allowing drug screening within weeks. In such techniques, the TME will be present in tissue-based cultures, but not from urine-derived cultures. Thus, both systems should be used complementary or supplemented with isolated cell line components to assess the TME contribution to disease and therapies. (3) both urinoid and tumoroid establishment and biobanking are time-consuming. This intrinsic limitation of patient-specific organoid research is being addressed using methods such as the previously mentioned rapid-organoid-based explant techniques.

Urinoids provide a unique opportunity to culture sequential follow-up samples from bladder cancer patients during their treatment. These insights will subsequently steer the development of current and novel combination therapies targeting drug-resistance pathways, or acquired vulnerabilities. Ultimately, urinoids are a non-invasive tool for following tumour pathogenesis, longitudinal drug-response monitoring, and therapy adaptation in bladder cancer.

### Supplementary information

Supplemental Figures and Tables

Supplemental Table 4

Supplemental Table 5

## Data Availability

The authors declare that the data supporting the findings of this study are available within the paper and its supplemental information files.

## Materials availability

Material and sequencing data supporting the findings of this study are available from the corresponding author upon reasonable request, according to the data protection assessment impact as part of the University Medical Center Utrecht (UMCU) FAIR principle under the Data Management Plan (DMP) ID#69968.
