# Supplementary material for: Urine-derived bladder cancer organoids (urinoids) as a tool for cancer longitudinal response monitoring and therapy adaptation
Source: Br J Cancer. 2023 Dec 15;130(3):369–79. doi: 10.1038/s41416-023-02494-6 (PMC10844626; doi:10.1038/s41416-023-02494-6)
Supplement: Supplementary file 1 — Supplemental Figures and Tables [file 41416_2023_2494_MOESM1_ESM.pdf]

Supplemental Figure 1. Immunohistochemical controls of bladder urinoids.

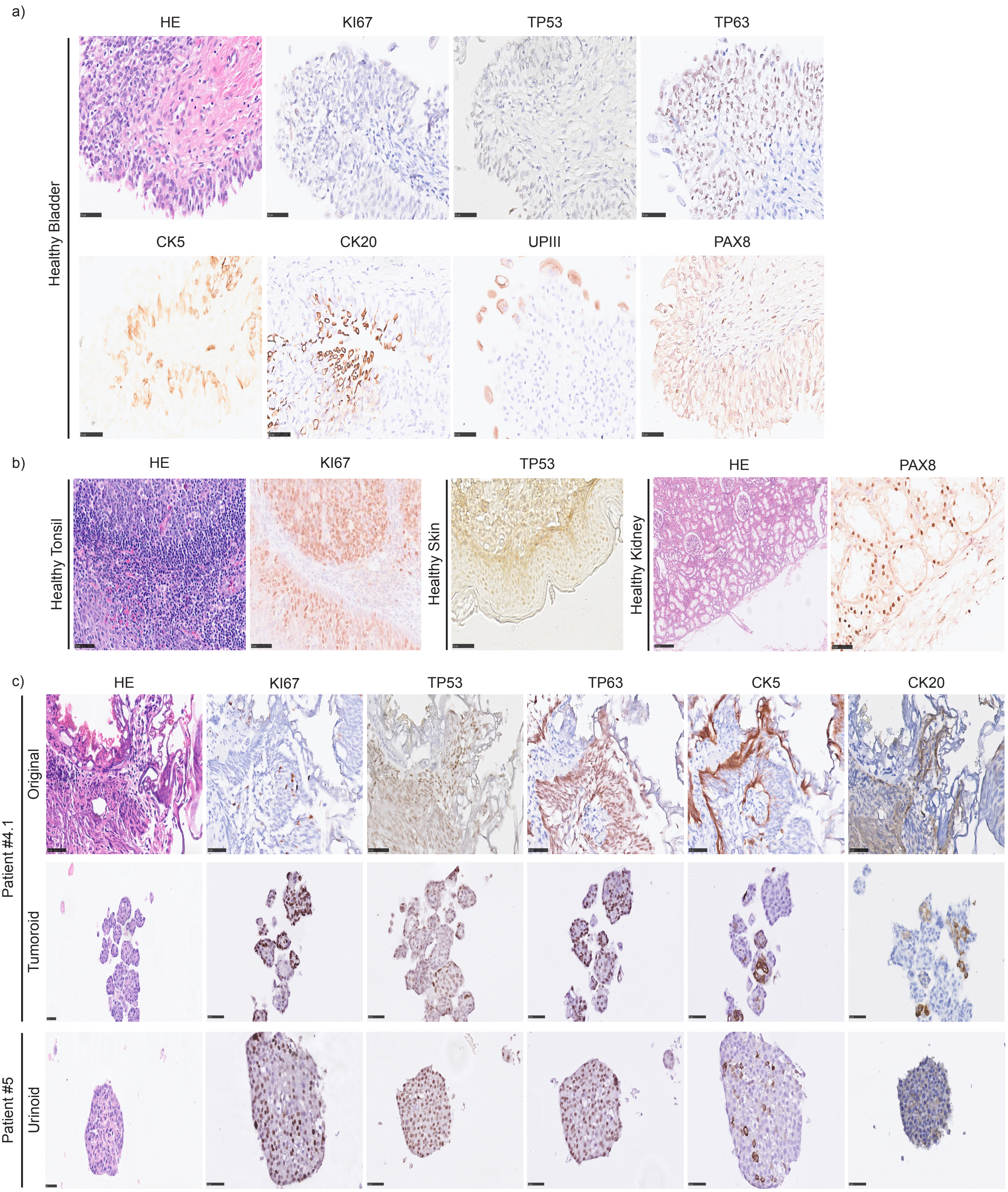

Supplemental Figure 1. Immunohistochemical controls of bladder urinoids.
Immunohistochemical analysis of (a) healthy bladder tissue displayed with hematoxylin and eosin (HE) and their expression of KI67, TP53, TP63, Keratin 5 (CK5), Keratin 20 (CK20), Uroplakin 3a (UPIII) & PAX8. (b) Positive control stainings for KI67, TP53 & PAX8 in Tonsil, Skin and Kidney tissue. (c) Immunohistochemical analysis of tumoroid 4.1 & UBTOR5 displayed with HE and the expression of Ki67, TP53, TP63, Keratin 5 (CK5) & Keratin 20 (CD20). Scale bars are 50 µm for tissues and organoids slides.

Supplemental Figure 2. Immunohistochemical analysis of UKPIII and PAX8.

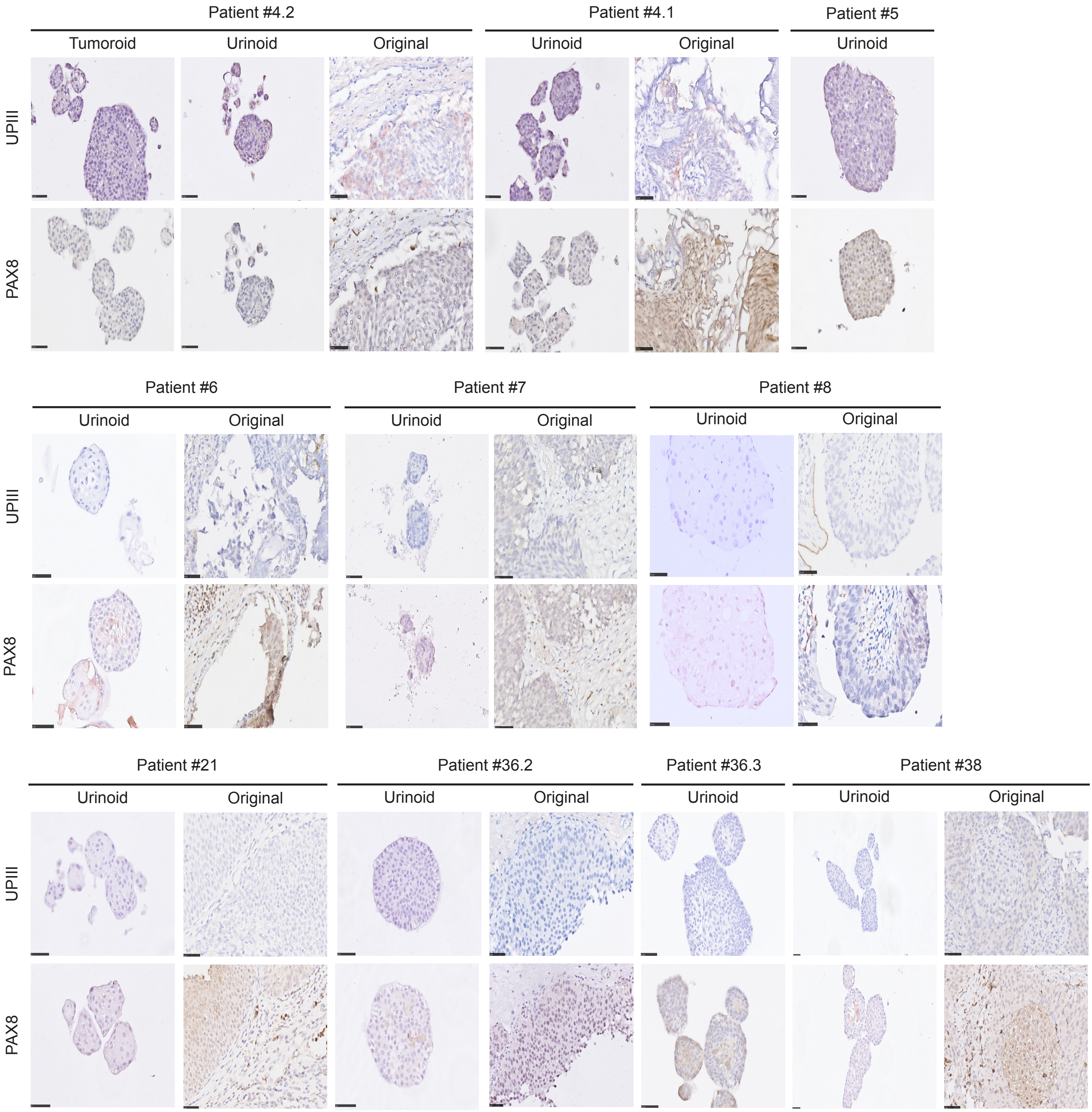

Supplemental Figure 2. Immunohistochemical analysis of UPIII and PAX8.
Immunohistochemical analysis of Original tumor tissues, urinoids and tumoroids displayed with their expression of Uroplakin 3a (UPIII) & PAX8. Scale bars are 50 μm for tissues and organoids slides.

Supplemental Figure 3. Whole genome sequencing of patient 4 urinoids & tumoroids.

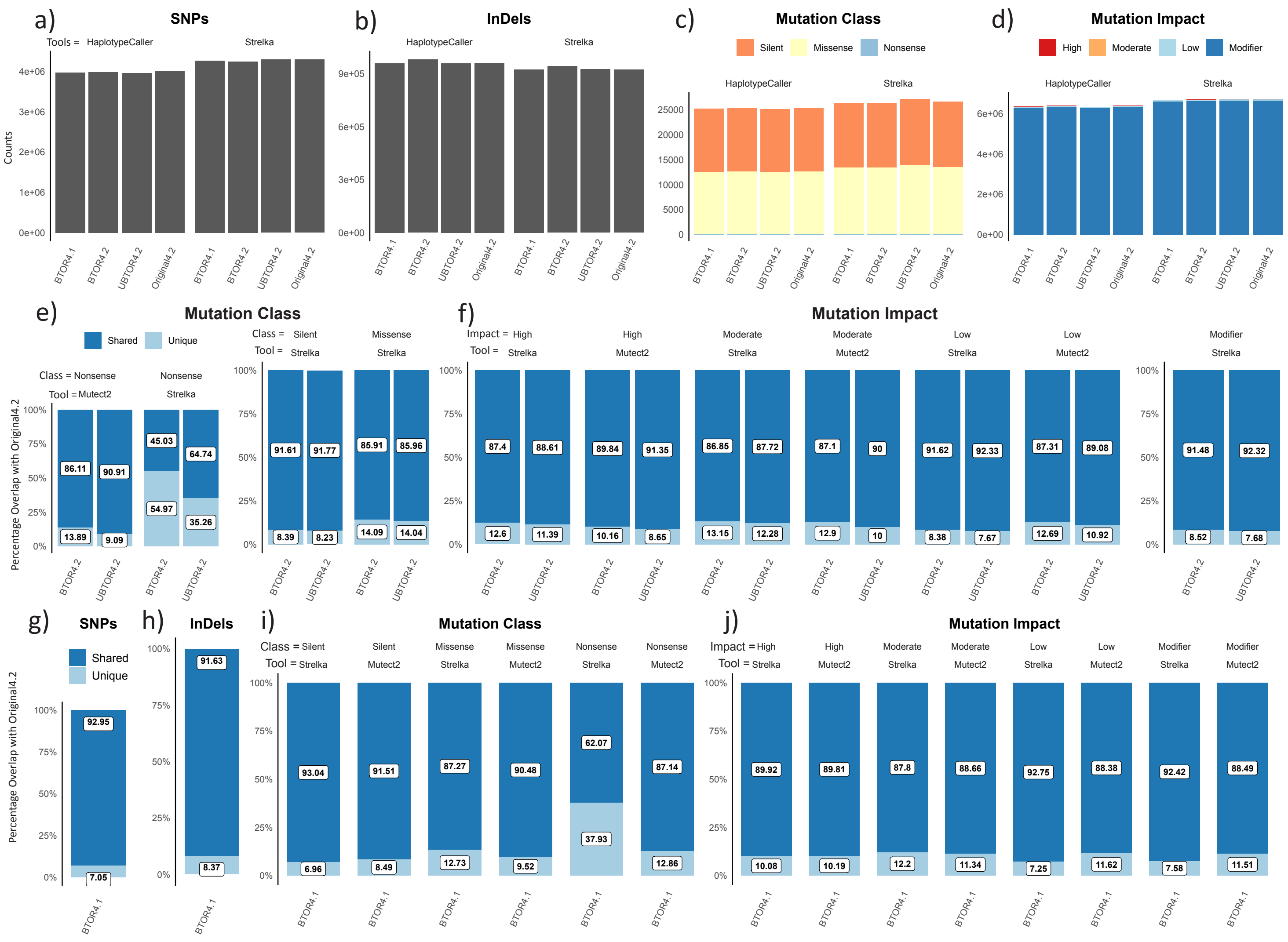

Supplemental Figure 3. Whole genome sequencing analysis of patient 4 urinoids & Tumoroids. Count of all patient 4 samples for (a) SNPs, (b) InDels, (c) Mutation class and (d) Mutation impact from the Sarek pipeline. Organoid correlation of mutations with the Original 4.2 sample for (e) nonsense (Mutect2 tool), silent and missense (Strelka tool) of mutation class and (f) high, moderate, low (Mutect2 & Strelka tools) and modifier (Strelka tool) impact of mutations for urinoids UBTOR4.2 and tumoroid BTOR4.2. Pre-treatment tumoroid BTOR4.1 correlation of mutations with the original 4.2 sample for (g) SNPs, (h) InDels, (i) mutation class and (j) mutation impact of Mutect2 and Strelka tools from the Sarek pipeline.

Supplemental Figure 4. Karyosequencing Patient 4.

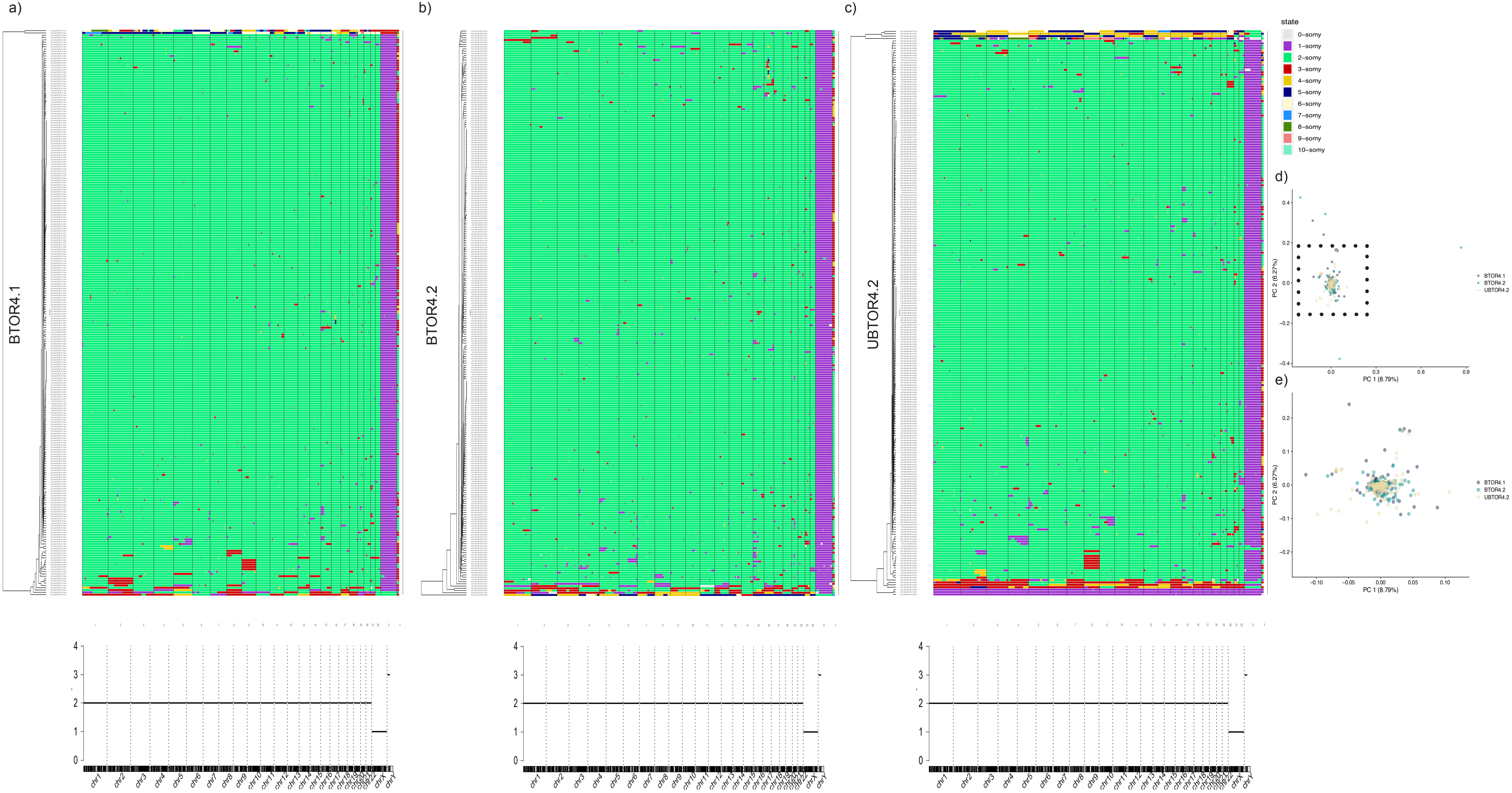

Supplemental Figure 4. Karyosequencing patient 4 organoids. Complete karyo-sequencing heatmaps of (a) BTOR4.1, (b) BTOR4.2 and (c) UBTOR4.2. All lines were single cell sorted and sequenced using single cell karyo-sequencing. Single cell data was analyzed using AneuFinder referenced to Genome Reference Consortium Human Build 38 from NCBI. Consensus ploidy plots of BTOR4.1, UBTOR4.2 and BTOR4.2 of patient four displayed in supplemental figure 5. (d) Principle component analysis of BTOR4.1, BTOR4.2 & UBTOR4.2 with (e) zoom of the densest region as indicated by the dashed box in.

Supplemental Figure 5. Immunotherapy induced structural variations in Tumoroids & Urinoids of Patient 4.

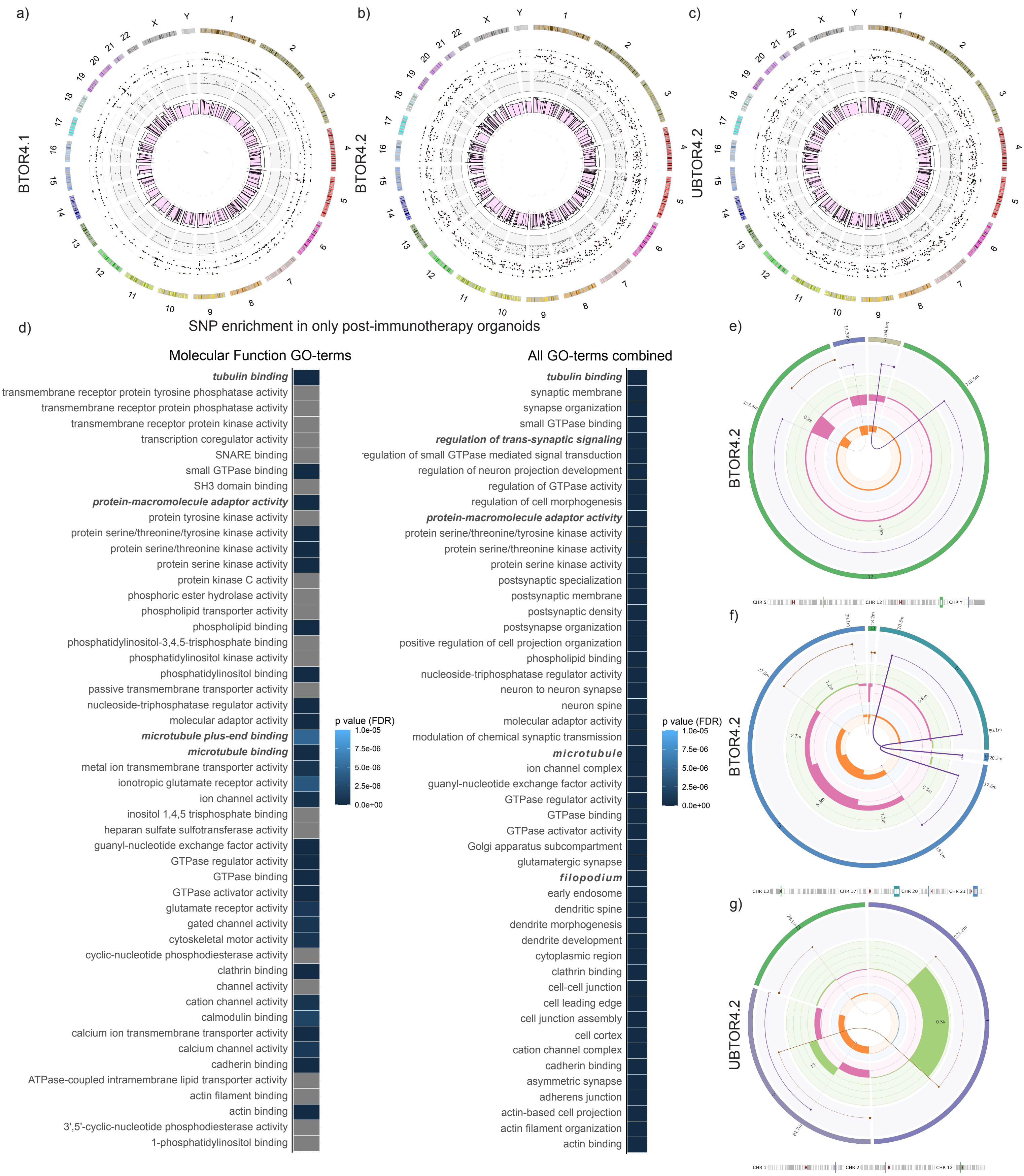

Supplemental Figure 5. Immunotherapy induced structural variations in tumoroids & urinoids of patient 4. Patient 4 circos overview plots from GRIDSS-PURPLE-LINX analysis with from the outside inwards the respective copy number variations (CNV), B-allele frequency (BAF), GC content and structural variations (SVs) interchromosomal (black) and intrachromosomal (red) variation of (a) BTOR4.1 tracks of pre-treatment tumoroid BTOR4.1 and post-treatment (b) tumoroid BTOR4.2 and (c) urinoid UBTOR4.2. (d) Highest quality scoring SNP enrichment terms (n=50) of SNPs only found in post-treatment organoids affecting genes associated with GO terms of Molecular Function (MF, left) and all Go terms (right), significance using false discovery rate (p<0.05) with bold highlighted terms are associated with microtubules. Complex multichromosomal linked structural variations as found by the LINX analysis of the GRIDSS-PURPLE-LINX pipeline for tumoroid (e) BTOR4.2 on chromosomes 5,12 & Y, (f) chromosomes 13,17, 20 & 21 and (g) urinoid UBTOR4.2 on chromosomes 1, 2 & 12.

Supplemental Figure 6. Application of urinoids in bladder cancer patients drug stratification.

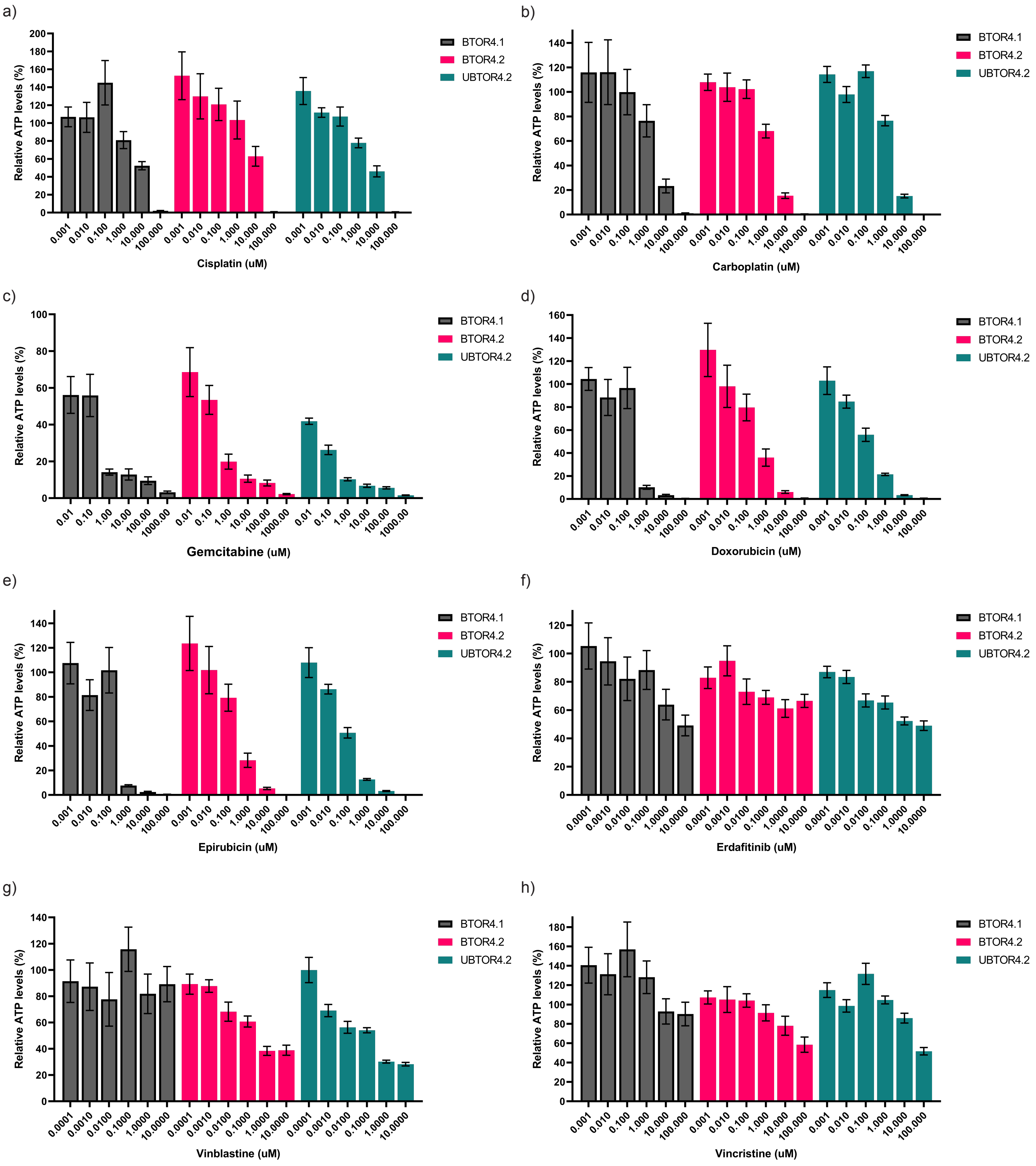

Supplemental Figure 6. Application of urinoids in bladder cancer patients drug stratification. Displayed are the relative ATP levels across a panel of common bladder cancer treatments for organoids BTOR4.1, BTOR4.2 & UBTOR4.2 for (a) Cisplatin, (b) Carboplatin, (c) Gemcitabin, (d) Doxorubicin, (e) Epirubicin, (f) Erdafitinib, (g) Vinblastine and (h) Vincristine. Displayed are the results of n = 6 replicates per drug concentration, consisting of 3 biological and 2 technical replicates with their respective s.e.m. deviation, normalized to no drug control condition (n = 6 per drug).

Supplemental Table 1. Summary of all patients included for patient-derived urinoid line establishment and corresponding clinical data

Patient #	Line	Classification	Subclassification	Gender	Age	Successful Establishment
4	UBTOR4.2	MIBC	ypT1G3 papillary UCC	M	65	Yes
5	UBTOR5	MIBC	TisN0MxR0	F	67	Yes
6	UBTOR6.1	NMIBC	pT1G3 papillary UCC	F	71	Yes
6	UBTOR6.2	NMIBC	pT1G3 papillary UCC	F	71	No
7	UBTOR7.1	NMIBC	pT1G3	M	73	Yes
8	UBTOR8	MIBC	pTisN0MxR0	M	58	Yes
9	UBTOR9	MIBC	pTisN0MxR0	M	76	No
15	UBTOR15	NMIBC	pT1G3	M	71	No
21	UBTOR21	MIBC	pT3N0MxR0 + SCC + PI	M	75	Yes
32	UBTOR32	NMIBC	pTaG1	M	75	No
36	UBTOR36.2	NMIBC	pT1G2-3	M	62	Yes
36	UBTOR36.3	NMIBC	pT1G2-3	M	62	Yes
38	UBTOR38	NMIBC	pTaG1-2	M	70	Yes
40	UBTOR40	MIBC	pT0N0	M	69	No
41	UBTOR41	MIBC	pT2bN0 UCC	M	74	Yes
42	UBTOR42	NMIBC	pTaG1	M	76	No
43	UBTOR43	NMIBC	pTisN0MxR0	M	68	No
44	UBTOR44	MIBC	pT0N0	M	72	No
45	UBTOR45	MIBC	pT2G3 UCC	M	60	Yes
50	UBTOR50	MIBC	cT3N1M1-ycT3N0M0 + SCC	F	54	No
51	UBTOR51	MIBC	pT3aN0MxR0 UCC	F	75	No
52	UBTOR52	MIBC	pT2G3 UCC	F	69	No
54	UBTOR54	NMIBC	pT0N0	M	76	No
57	UBTOR57	NMIBC	pT1G3 Papillary UCC	M	71	Yes
61	UBTOR61	NMIBC	pTisN0MxR0	M	74	No

Subclassification given based on TNM classification and pathologist report

UCC = Urothelial carcinoma; SCC = Squamous cell carcinoma; PI = Peritoneal invasion

Supplemental Table 2. Summary immunohistochemical results original tumor tissues and organoid

Patient #	Original / Line	Molecular subtype estimation
4	Original Tumor Tissue	Transitional / Intermediate & luminal / luminal-like subtype
	UBTOR4.2	Transitional / Intermediate & luminal / luminal-like subtype
5	Original Tumor Tissue	n.a.
	UBTOR5	Transitional / Intermediate & luminal / luminal-like subtype
6	Original Tumor Tissue	Basal / Basal-like subtype
	UBTOR6.1	Basal / Basal-like subtype
7	Original Tumor Tissue	Basal / Basal-like subtype
	UBTOR7.1	Basal / Basal-like subtype
8	Original Tumor Tissue	Transitional / Intermediate & luminal / luminal-like subtype
	UBTOR8	Transitional / Intermediate & luminal / luminal-like subtype
21	Original Tumor Tissue	Basal / Basal-like subtype
	UBTOR21	Basal / Basal-like subtype
36	Original Tumor Tissue	Basal / Basal-like subtype
	UBTOR36.2	Basal / Basal-like subtype
	UBTOR36.3	Basal / Basal-like subtype
38	Original Tumor Tissue	Basal / Basal-like subtype
	UBTOR38	Basal / Basal-like subtype
41	Original Tumor Tissue	Basal / Basal-like subtype
	UBTOR41	Basal / Basal-like subtype
45	Original Tumor Tissue	Basal / Basal-like subtype
	UBTOR45	Basal / Basal-like subtype
57	Original Tumor Tissue	Basal / Basal-like subtype
	UBTOR57	Basal / Basal-like subtype

Molecular subtypes are estimated on the current consensus classifications; CytoKeratin 5 (CK5) positive samples = luminal or luminal-like subtype, CytoKeratin 20 (CK20) positive samples = basal or basal-like subtype, tumor protein 63 (TP63) positive samples = intermediate subtype. Original tumor sample patient 5 was lost due to a clerical error at the pathology, thus not available for analysis.

Supplemental Table 3. Fusion proteins of BTOR4.1, BTOR4.2 and UBTOR4.2 detected by Manta

Organoid Line	Fusion Gene Name	ReportedType	Phased	GeneStart	GeneContextStart	TranscriptStart	GeneEnd	GeneContextEnd	TranscriptEnd
BTOR4.1	NUGGC_SCARA5	NONE	INFRAME	NUGGC	Exon 18	ENST00000413272	SCARA5	Promoter Region	ENST00000518030
BTOR4.1	CHD7_ZC3H12A	NONE	INFRAME	CHD7	Exon 2	ENST00000423902	ZC3H12A	Promoter Region	ENST00000471012
BTOR4.1	POLRMT_SYPL1	NONE	SKIPPED_EXONS	POLRMT	Exon 1	ENST00000592863	SYPL1	Exon 6	ENST00000470347
BTOR4.1	EXT2_SYPL1	NONE	SKIPPED_EXONS	EXT2	Exon 11	ENST00000533608	SYPL1	Exon 3	ENST00000011473
BTOR4.1	SPTBN1_SPTBN1	NONE	OUT_OF_FRAME	SPTBN1	Exon 2	ENST00000356805	SPTBN1	Exon 4	ENST00000356805
BTOR4.1	BTG3_TBC1D16	NONE	INFRAME	BTG3	Exon 4	ENST00000339775	TBC1D16	Promoter Region	ENST00000574241
BTOR4.1	KMT2D_KMT2D	NONE	OUT_OF_FRAME	KMT2D	Exon 11	ENST00000301067	KMT2D	Exon 11	ENST00000301067
BTOR4.1	PHACTR4_PHACTR4	NONE	OUT_OF_FRAME	PHACTR4	Exon 3	ENST00000632421	PHACTR4	Exon 2	ENST00000632421
BTOR4.1	NINL_NINL	NONE	INFRAME	NINL	Exon 17	ENST00000278886	NINL	Exon 17	ENST00000278886
BTOR4.1	FGFR3_TACC3	KNOWN_PAIR	INFRAME	FGFR3	Exon 17	ENST00000340107	TACC3	Exon 8	ENST00000313288
BTOR4.1	AC113348.2_CLK4	NONE	SKIPPED_EXONS	AC113348.2	Exon 4	ENST00000638723	CLK4	Promoter Region	ENST00000316308
BTOR4.2	NUGGC_SCARA5	NONE	INFRAME	NUGGC	Exon 18	ENST00000413272	SCARA5	Promoter Region	ENST00000518030
BTOR4.2	SPTBN1_SPTBN1	NONE	OUT_OF_FRAME	SPTBN1	Exon 2	ENST00000356805	SPTBN1	Exon 4	ENST00000356805
BTOR4.2	BTG3_TBC1D16	NONE	INFRAME	BTG3	Exon 4	ENST00000339775	TBC1D16	Promoter Region	ENST00000574241
BTOR4.2	KMT2D_KMT2D	NONE	OUT_OF_FRAME	KMT2D	Exon 11	ENST00000301067	KMT2D	Exon 11	ENST00000301067
BTOR4.2	NINL_NINL	NONE	INFRAME	NINL	Exon 17	ENST00000278886	NINL	Exon 17	ENST00000278886
BTOR4.2	FGFR3_TACC3	KNOWN_PAIR	INFRAME	FGFR3	Exon 17	ENST00000340107	TACC3	Exon 8	ENST00000313288
BTOR4.2	ADGRB3_ADGRB3	NONE	OUT_OF_FRAME	ADGRB3	Exon 8	ENST00000370598	ADGRB3	Exon 11	ENST00000370598
BTOR4.2	AC113348.2_CLK4	NONE	SKIPPED_EXONS	AC113348.2	Exon 4	ENST00000638723	CLK4	Promoter Region	ENST00000316308
UBTOR4.2	NUGGC_SCARA5	NONE	INFRAME	NUGGC	Exon 18	ENST00000413272	SCARA5	Promoter Region	ENST00000518030
UBTOR4.2	ZNF236_TRAF3	NONE	SKIPPED_EXONS	ZNF236	Exon 30	ENST00000543926	TRAF3	Exon 2	ENST00000560371
UBTOR4.2	SPTBN1_SPTBN1	NONE	OUT_OF_FRAME	SPTBN1	Exon 2	ENST00000356805	SPTBN1	Exon 4	ENST00000356805
UBTOR4.2	ARFGAP3_ACYP2	NONE	SKIPPED_EXONS	ARFGAP3	Exon 7	ENST00000263245	ACYP2	Promoter Region	ENST00000406041
UBTOR4.2	KMT2D_KMT2D	NONE	OUT_OF_FRAME	KMT2D	Exon 11	ENST00000301067	KMT2D	Exon 11	ENST00000301067
UBTOR4.2	BTG3_TBC1D16	NONE	INFRAME	BTG3	Exon 4	ENST00000339775	TBC1D16	Promoter Region	ENST00000574241
UBTOR4.2	PHACTR4_PHACTR4	NONE	OUT_OF_FRAME	PHACTR4	Exon 3	ENST00000632421	PHACTR4	Exon 2	ENST00000632421
UBTOR4.2	FGFR3_TACC3	KNOWN_PAIR	INFRAME	FGFR3	Exon 17	ENST00000340107	TACC3	Exon 8	ENST00000313288

All fusion proteins found in BTOR4.1, BTOR4.2 and UBTOR4.2 using Manta analysis. Known fusion pairs are indicated by KNOWN_PAIR.
